# Characterization of the DNAM-1, TIGIT and TACTILE Axis on Circulating NK, NKT-Like and T Cell Subsets in Patients with Acute Myeloid Leukemia

**DOI:** 10.3390/cancers12082171

**Published:** 2020-08-05

**Authors:** Isabel Valhondo, Fakhri Hassouneh, Nelson Lopez-Sejas, Alejandra Pera, Beatriz Sanchez-Correa, Beatriz Guerrero, Juan M. Bergua, Maria Jose Arcos, Helena Bañas, Ignacio Casas-Avilés, Joaquin Sanchez-Garcia, Josefina Serrano, Carmen Martin, Esther Duran, Corona Alonso, Rafael Solana, Raquel Tarazona

**Affiliations:** 1 Immunology Unit, University of Extremadura, 10003 Cáceres, Spain; ivalhondog@gmail.com (I.V.); hassounehfakhri@yahoo.com (F.H.); nelsonj836@hotmail.com (N.L.-S.); beatrizsanchezcorrea@gmail.com (B.S.-C.); beatrizguerrerosimon@gmail.com (B.G.); rtarazon@unex.es (R.T.); 2 Department of Immunolgy and Allergy, Instituto Maimónides de Investigación Biomédica de Córdoba (IMIBIC), 14004 Cordoba, Spain; alejandrapera@gmail.com; 3 Department of Hematology, Hospital San Pedro de Alcantara, 10003 Caceres, Spain; jmberguaburg@gmail.com (J.M.B.); mjarcar@yahoo.es (M.J.A.); cromacita@gmail.com (H.B.); nachocasas@hotmail.com (I.C.-A.); 4 Department of Hematology, Reina Sofia University Hospital, 14004 Córdoba, Spain; joaquin.sanchez@cheerful.com (J.S.-G.); josefina.serrano@iname.com (J.S.); carmen.martin.calvo.sspa@juntadeandalucia.es (C.M.); 5 Histology and Pathology Unit, Faculty of Veterinary, University of Extremadura, 10003 Cáceres, Spain; esther@unex.es; 6 Department of Immunology and Allergology, Reina Sofia University Hospital, 14004 Córdoba, Spain; 7 Department of Cell Biology, Physiology and Immunology, University of Cordoba, 14004 Córdoba, Spain

**Keywords:** acute myeloid leukemia, DNAM-1, NK cells, NKT-like cells, TACTILE, T cells, TIGIT

## Abstract

*Background*: Acute myeloid leukemia (AML) remains a major clinical challenge due to poor overall survival, which is even more dramatic in elderly patients. TIGIT, an inhibitory receptor that interacts with CD155 and CD112 molecules, is considered as a checkpoint in T and NK cell activation. This receptor shares ligands with the co-stimulatory receptor DNAM-1 and with TACTILE. The aim of this work was to analyze the expression of DNAM-1, TIGIT and TACTILE in NK cells and T cell subsets in AML patients. *Methods*: We have studied 36 patients at the time of diagnosis of AML and 20 healthy volunteers. The expression of DNAM-1, TIGIT and TACTILE in NK cells and T cells, according to the expression of CD3 and CD56, was performed by flow cytometry. *Results*: NK cells, CD56− T cells and CD56+ T (NKT-like) cells from AML patients presented a reduced expression of DNAM-1 compared with healthy volunteers. An increased expression of TIGIT was observed in mainstream CD56− T cells. No differences were observed in the expression of TACTILE. Simplified presentation of incredibly complex evaluations (SPICE) analysis of the co-expression of DNAM-1, TIGIT and TACTILE showed an increase in NK and T cells lacking DNAM-1 and co-expressing TIGIT and TACTILE. Low percentages of DNAM-1−TIGIT+TACTILE+ NK cells and DNAM-1− TIGIT+TACTILE+ CD56− T cells were associated with a better survival of AML patients. *Conclusions*: The expression of DNAM-1 is reduced in NK cells and in CD4+ and CD8+ T cells from AML patients compared with those from healthy volunteers. An increased percentage of NK and T cells lacking DNAM-1 and co-expressing TIGIT and TACTILE is associated with patient survival, supporting the role of TIGIT as a novel candidate for checkpoint blockade.

## 1. Introduction

Acute myeloid leukemia (AML) is a hematopoietic malignancy characterized by a clonal expansion of low differentiated hematopoietic precursors that infiltrate the bone marrow and limit normal hematopoiesis. Although AML occurs in all ages, it appears predominantly in older people (>60 years of age). AML is characterized by a poor prognosis and management difficulties, which is even more difficult in elderly patients who frequently have other pathologies associated with age [1]. 

The capacity of the immune system to control tumor growth and metastasis has focused attention in order to develop new strategies to treat cancer directed to stimulate the patient immune system such as checkpoint blockade or using engineered immune cells to attack cancer as it is currently used in CAR-T therapies. Thus, novel cancer immunotherapies have emerged in the last decade that are being analyzed in clinical trials in patients with hematologic and solid cancer [2,3,4]. 

The analysis of the patient immune system represents a central point for the design of personalized immunotherapies. Several alterations have been described in T and NK cells in AML patients that limit tumor control by the immune system [2,5,6].

Nectin and nectin-like molecules can represent biomarkers for cancer diagnosis, players in immune responses and targets for cancer immunotherapy [7,8,9,10,11]. DNAM-1 (also known as CD226) was first discovered as a costimulatory receptor expressed on cytotoxic T cells [12]. DNAM-1 is also expressed by NK cells and is involved in T cell- and NK cell-mediated cytotoxicity [13]. CD112 (Nectin-2) and CD155 (Necl-5) were identified as ligands for the DNAM-1 receptor [11]. DNAM-1 crosslinking induces Akt phosphorylation, and its co-engagement with CD244 (also termed 2B4), induces the phosphorylation of Erk, phosphorylation of p65 and NF-kB activation [14]. We have previously shown a reduced expression of DNAM-1 on NK cells from AML patients that correlates with CD112 expression on AML blasts. In vitro experiments demonstrated that DNAM-1 is downregulated after contact with leukemic blasts positive for DNAM-1 ligands (CD112 and CD155) [5,6]. A decreased expression of DNAM-1 has also been reported in T cells from AML patients that also correlated with CD112 expression on leukemic blasts [15,16]. 

In the last decade, one of the major advances in cancer immunotherapy was the use of monoclonal antibodies (mAbs) to block inhibitory immune checkpoints such as PD-1 and CTLA-4. Recently, the landscape of checkpoint blockade therapies has incorporated novel checkpoints that contribute, at least in part, to the immunosuppressive milieu that is frequently observed in cancer patients. One of these inhibitory receptors is TIGIT, that is expressed in different immune cells such as T cells, regulatory T cells, follicular T cells and NK cells [17,18,19]. TIGIT shares the same ligands, CD155 and CD112, with the activating/co-stimulatory receptor DNAM-1 and shows higher affinity than DNAM-1 for CD155 [20,21]. 

TACTILE (also known as CD96) shares the CD155 ligand with TIGIT and DNAM-1. TACTILE is expressed in T and NK cells. In mouse, TACTILE acts as an inhibitory receptor. Conversely, in humans TACTILE possesses both inhibitory and activating motives, and its implication in the regulation of lymphocyte activation is not fully understood [22]. 

Considering that DNAM-1, TIGIT and TACTILE constitute an axis of paired receptors with opposite functions on NK and T cells, that are key regulators of immune surveillance after recognition of their ligands on tumor cells, (for review see [21]) and that antibodies to TIGIT are now included in clinical trials in cancer patients, a better knowledge of the expression of these receptors on T and NK lymphocytes in healthy individuals and AML patients is required for a better understanding of the mechanisms involved in cancer escape from immune effector cells. Thus, the aim of this study was to analyze the expression of DNAM-1, TIGIT and TACTILE in AML patients compared with healthy volunteers. We have analyzed the expression of these receptors in a cohort of AML patients in NK cells, conventional T cells (CD3+CD56−) and NKT-like (CD3+CD56+) cells. Boolean analysis of the expression of DNAM-1, TIGIT and TACTILE phenotypes was performed using simplified presentation of incredibly complex evaluations (SPICE) analysis.

## 2. Results

### 2.1. Peripheral Blood Distribution of NK and T Cells in AML Patients at Diagnostic

The analysis of NK cell percentage showed a significant decrease in AML patients when compared with healthy donors (*p* = 0.003) (Table 1). In contrast, no statistically significant differences were observed in the percentage of T cells independently of CD56 expression (Table 1). 

### 2.2. Expression of DNAM-1, TIGIT and TACTILE Receptors in NK and T Cells

**Figure 1:**
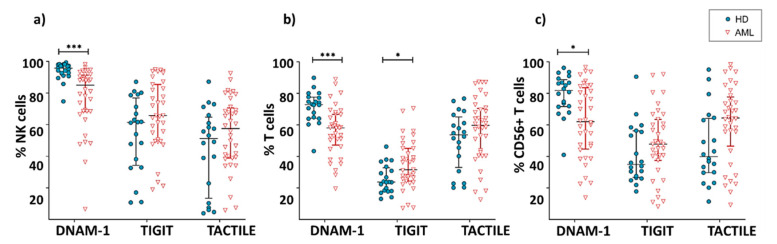
Expression of DNAM-1, TIGIT and TACTILE. Percentage of NK cells (**a**), conventional CD56− T cells (**b**) and CD56+ NKT-like cells (**c**) expressing DNAM-1, TIGIT and TACTILE in AML patients (*n* = 36) and HD (*n* = 20). Vertical lines indicate interquartile ranges from the 25th to the 75th percentile. The horizontal lines represent the median values. Results were considered significant at * *p* = 0.02 and *** *p* < 0.001. HD: healthy donors, AML: acute myeloid leukemia patients.

DNAM-1 was expressed in both NK and T cells, with NK cells being the population with the highest percentage of expression of this receptor (Figure 1). The comparison between AML patients and healthy donors showed a significant decrease (*p* < 0.001) in the percentage of DNAM-1+ NK cells in AML patients (AML: 76.8 ± 20.4%; HD: 93.9 ± 5.9%) (Figure 1a). AML patients also showed a significant decrease in the expression of DNAM-1 in conventional CD56− T cells (56 ± 11.8% in AML vs. 71.7 ± 9.7 in healthy donors; *p* < 0.001) and CD56+ NKT-like cells (65.9 ± 20.6% in AML vs. 79 ± 13.8 in healthy donors; *p* = 0.02) (Figure 1b,c).

The inhibitory receptor TIGIT was expressed in a high percentage of NK cells. In T cells, the percentage of TIGIT+ cells was higher within T cells expressing CD56 than in their CD56- counterpart (Figure 1). When comparing TIGIT expression between AML patients and healthy donors, no significant differences were found within NK cells (61.2 ± 19.9% vs. 50.4 ± 24.6%, respectively) or CD56+ T cells (45.1 ± 21.1% vs. 36.9 ± 19.9%, respectively). Conversely, the percentage of TIGIT+ CD56- T cells was significantly higher (*p* = 0.02) in AML patients (32.3 ± 14.9%) than in healthy donors (23.3 ± 8.9%).

When the expression of TACTILE was analyzed on AML and healthy donors, no significant differences were found in NK (48.4 ± 22.6% vs. 46.3 ± 26.7%, respectively), conventional T cells (48.3 ± 20.8% vs. 51.1 ± 17.1%) or CD56+ NKT-like cells (55.7 ± 25.8% vs. 45.4 ± 22.3%) (Figure 1). 

### 2.3. Boolean Analysis of the Co-Expression of DNAM-1, TIGIT and TACTILE in NK and T Cells

**Figure 2:**
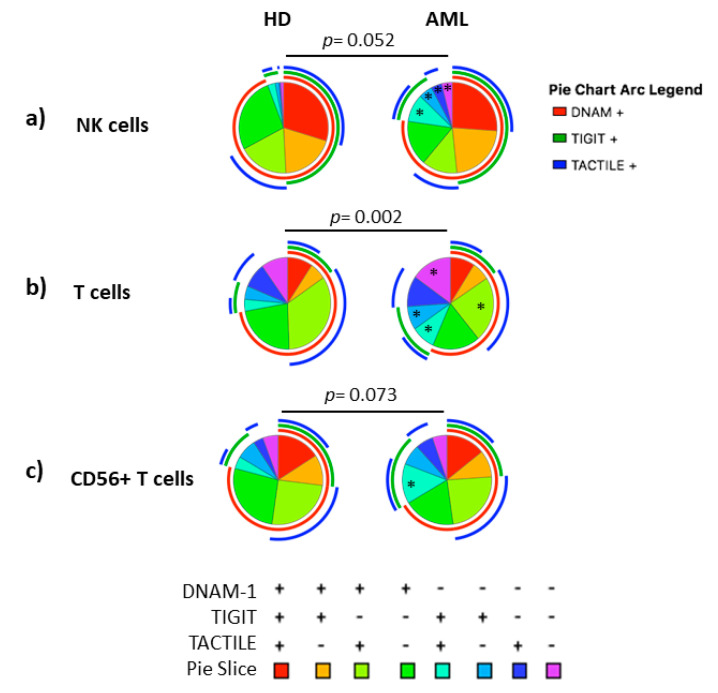
Co-expression patterns (pie charts) of DNAM-1, TIGIT and TACTILE analyzed in (**a**) NK cells, (**b**) conventional CD56− T cells and (**c**) CD56+ NKT-like cells from healthy individuals (*n* = 20) and AML patients (*n* = 30). Positive and negative expression of DNAM-1, TIGIT and TACTILE were combined by Boolean gating to generate all possible subsets. Each color in the pie corresponds to specific combination of antigens indicated in the bottom part of the figure. The asterisk (*) within the slices refers to statistically significant differences between AML patients and healthy donors for the indicated subsets (*p* < 0.05). HD: healthy donors, AML: acute myeloid leukemia patients.

The co-expression patterns of DNAM-1, TIGIT and TACTILE receptors in NK cells, conventional CD56− T cells and CD56+ NKT-like cells from healthy individuals and AML patients gated using Boolean analysis as indicated in Materials and Methods are shown in Figure 2. Eight different possible phenotype combinations were analyzed, and phenotype profiles were analyzed by the SPICE software.

**Figure 3:**
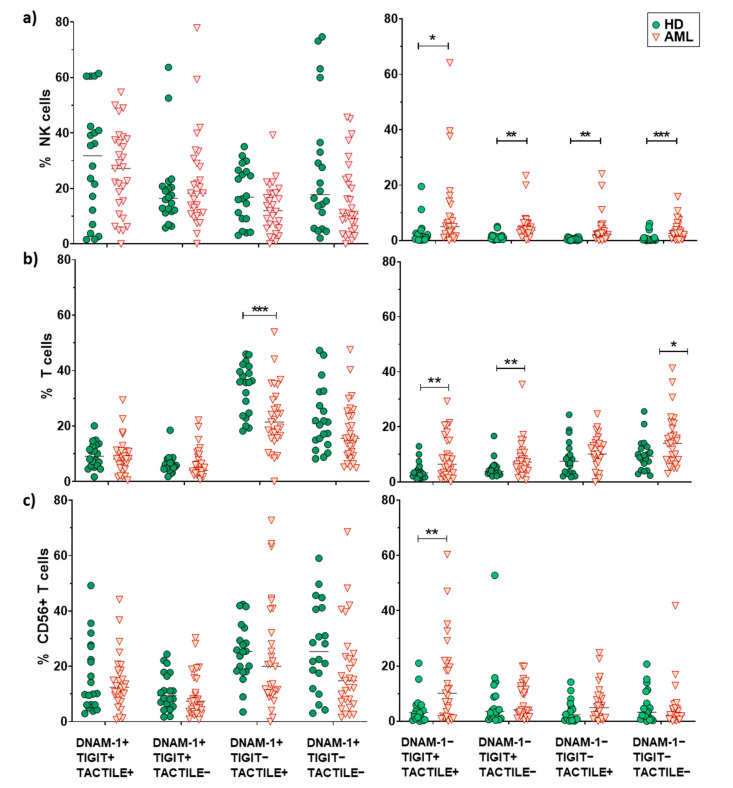
Analysis of DNAM-1, TIGIT and TACTILE co-expression. Eight different subpopulations can be observed according to the co-expression of DNAM-1, TIGIT and TACTILE. The distribution of these subsets in NK cells (**a**), CD56− T cells (**b**) and CD56+ T cells (**c**) is shown. The median values are indicated by a horizontal black line. Results were considered significant at * *p* < 0.05 and ** *p* < 0.01 *** *p* < 0.001.

No statistically significant differences (*p* = 0.052) were found when comparing the receptor expression profiles in NK cells from AML patients and healthy donors (pie charts) (Figure 2a). Nevertheless, when each combination was analyzed independently, AML patients showed a significantly higher percentage of DNAM-1−TIGIT+TACTILE+ (*p* = 0.02), DNAM-1−TIGIT+TACTILE− (*p* = 0.001), DNAM-1−TIGIT−TACTILE+ (*p* = 0.003) and DNAM-1−TIGIT−TACTILE− (*p* = 0.001) NK cell subsets, compared to healthy donors (Figure 2a and Figure 3a). 

The co-expression profile of DNAM-1, TIGIT and TACTILE on conventional CD56− T cells from AML patients was significantly different than the profile observed on healthy donors (*p* = 0.002), (Figure 2b). The analysis of the specific subsets showed that AML patients had a significant decrease in the percentage of DNAM-1+TIGIT−TACTILE+ T cells (*p* = 0.001) and an increase in the percentage of DNAM-1−TIGIT+TACTILE+ (*p* = 0.004), DNAM-1−TIGIT+TACTILE− (*p* = 0.02) and DNAM-1− TIGIT− TACTILE− (*p* = 0.02) T cells (Figure 2b and Figure 3b). 

The analysis of CD56+ NKT-like cells did not show statistically significant differences in the pattern distribution in the pie chart in AML patients compared with healthy donors (Figure 2c). When comparing each combination independently, the only significant difference observed was an increase in the percentage of DNAM-1−TIGIT+TACTILE+ NKT-like cells (*p* = 0.002) in AML patients (Figure 2c and Figure 3c). 

When comparing the expression profiles among subsets, significant differences were observed between NK cells and T cells (*p* < 0.0001 in both healthy donors and AML patients) and between NK cells and CD56+ NKT-like cells (*p* = 0.001 in healthy donors and *p* = 0.03 in AML patients). The distribution of the pie chart also differed between T cells and CD56+ T cells (*p* = 0.01 in both healthy donors and AML patients) (Figure S1).

### 2.4. Expression of DNAM-1, TIGIT and TACTILE on CD4 and CD8 T Cell Subsets

**Figure 4:**
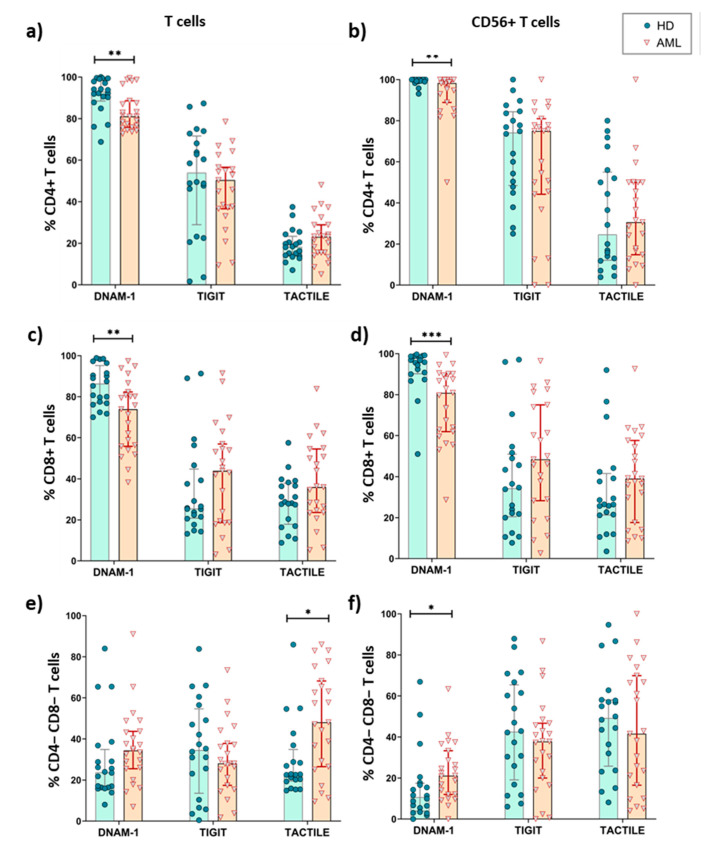
Expression of DNAM-1, TACTILE and TIGIT in T cells subsets from AML patients and healthy donors. The percentages of CD4+ (**a**,**b**), CD8+ (**c**,**d**) and CD4−CD8− (**e**,**f**) T cells expressing DNAM-1, TACTILE and TIGIT were analyzed in conventional CD56− conventional T cells (**a**,**c**,**e**) and CD56+ NKT-like cells (**b**,**d**,**f**) in AML patients (*n* = 23) and HD (*n* = 20). Vertical lines indicate interquartile ranges, from the 25th to the 75th percentile. The horizontal lines represent the median values. * *p* < 0.05, ** *p* < 0.01, *** *p* < 0.001. HD: healthy donors, AML: acute myeloid leukemia.

We also investigated whether the expression of DNAM-1, TIGIT and TACTILE differed among conventional CD56- T cells and CD56+ NKT-like cells subsets defined by CD4 and CD8 expression (Table 1, Figure 4). As the percentage of double positive (DP) CD4+CD8+ T cells was very low, both in AML patients and healthy donors, these cells were excluded in further analyses. In both healthy donors and AML patients, double negative (DN) CD4-CD8- T cells showed the lowest percentages of DNAM-1+ cells independently of CD56 expression and CD4+CD56+ T cells the highest expression. 

When comparing between AML patients and healthy donors, the results show that the percentage of CD4+ and CD8+ T cells expressing DNAM-1+ was significantly reduced in AML patients, whereas the percentages of TACTILE+ cells and TIGIT+ cells were preserved (Figure 4a–d). The percentages of TACTILE+ DN T cells (Figure 4e) and DNAM-1+ CD56+ DN T cells (Figure 4f) were increased in AML patients. 

### 2.5. Boolean Analysis of the Co-Expression of DNAM-1, TIGIT and TACTILE on CD4 and CD8 T Cell Subsets

**Figure 5:**
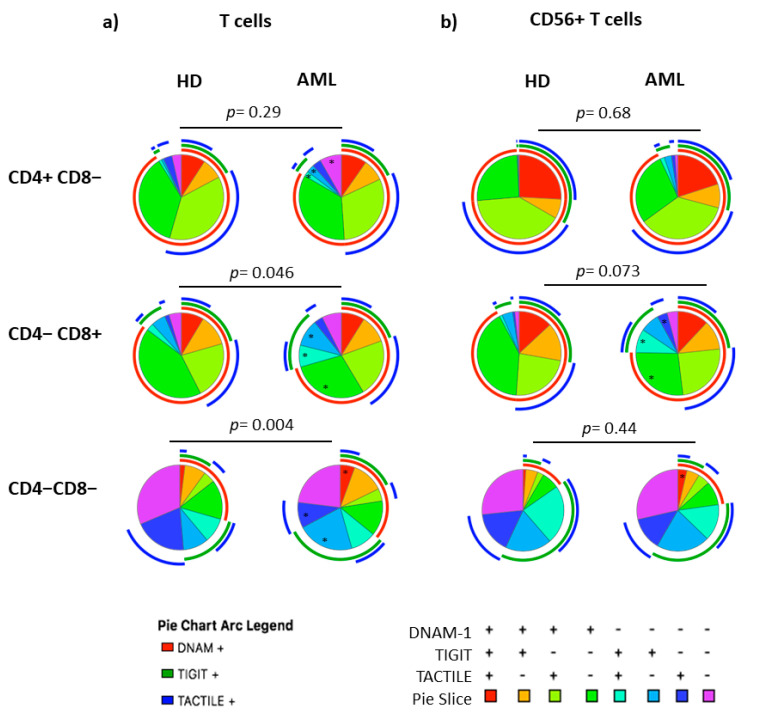
Co-expression patterns (pie charts) of DNAM-1, TIGIT and TACTILE in CD4+, CD8+ and DN T cell subsets distributed according to CD56 expression: conventional CD56− T (**a**) and CD56+ NKT-like (**b**) cells from HD (*n* = 20) and AML patients (*n* = 23). The asterisk (*) within the slices refers to statistically significant differences between AML patients and healthy donors for the indicated subsets (*p* < 0.05). HD: healthy donors, AML: acute myeloid leukemia patients.

**Figure 6:**
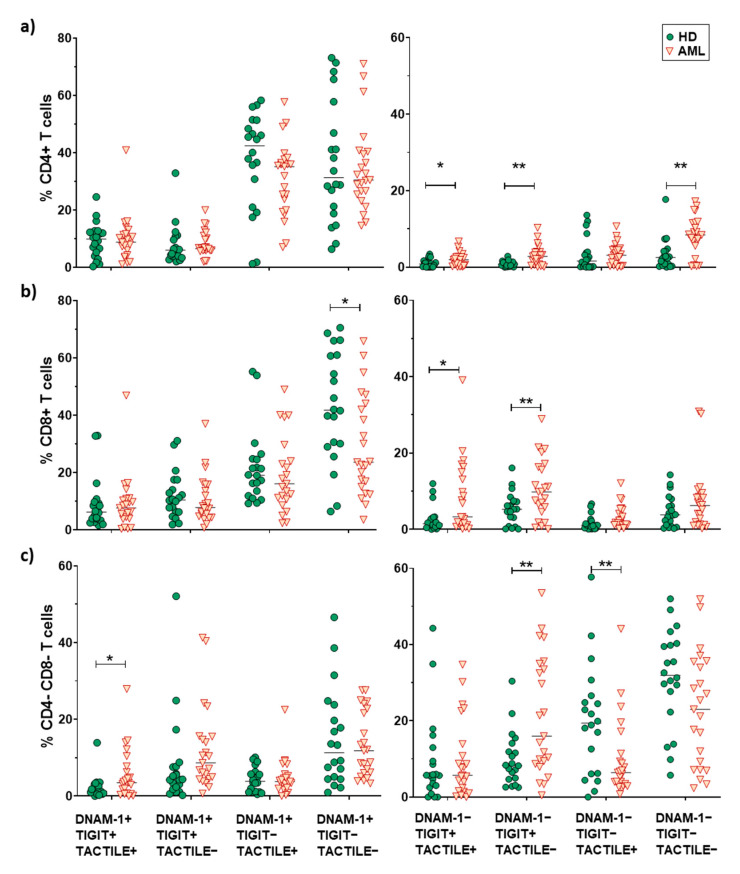
Analysis of DNAM-1, TIGIT and TACTILE co-expression in CD4+, CD8+ and CD4−CD8− T cell subsets. Eight different subpopulations can be observed according to the co-expression of DNAM-1, TIGIT and TACTILE. The distribution of these subsets in CD4+ T cells (**a**), CD8+ T cells (**b**) and CD4−CD8− T cells (**c**) in HD (*n* = 20) and AML patients (*n* = 23) is shown. The median values are indicated by a horizontal black line. Results were considered significant at * *p* < 0.05 and ** *p* < 0.01.

The co-expression patterns of DNAM-1, TIGIT and TACTILE receptors on CD4 and CD8 T cell subsets cells from healthy individuals and AML patients, gated using Boolean analysis as indicated in Materials and Methods, are shown in Figure 5. The study of CD4+ T cells pie distribution showed no significant differences between AML patients and healthy donors (Figure 5a, upper panel). However, when looking at each combination independently (pie slices), an increase in the percentage of DNAM-1−TIGIT+TACTILE+ cells (*p* = 0.036), DNAM-1−TIGIT+TACTILE−cells (*p* = 0.002) and DNAM-1−TIGIT−TACTILE− cells (*p* = 0.002) was observed in AML patients (Figure 5a, upper row and Figure 6a). 

The analysis of CD8+ T cell subset pie distribution (Figure 5a, middle row) showed statistically significant differences between AML patients and healthy donors (*p* = 0.046). Specifically, DNAM-1+TIGIT−TACTILE− cells (*p* = 0.023) were reduced in AML patients, while DNAM-1−TIGIT+TACTILE+ cells (*p* = 0.014) and DNAM-1− TIGIT+TACTILE− cells (*p* = 0.01) were increased (Figure 5a, middle row and Figure 6b). 

The analysis of DNAM-1, TIGIT and TACTILE co-expression in DN T cell subset (Figure 5a, lower row) also showed statistically significant differences in the distribution of pie charts between AML patients and healthy donors (*p* = 0.004) (Figure 5a, lower row). In healthy donors, the percentage of DNAM-1+TIGIT+TACTILE+ DN T cells was negligible. An increase in DNAM-1+TIGIT+TACTILE+ (*p* < 0.05) and DNAM-1−TIGIT+TACTILE− (*p* < 0.01) DN T cells and a decrease in DNAM-1− TIGIT− TACTILE+ (*p* < 0.01) DN T cells were observed in AML patients compared to healthy donors (Figure 6c).

**Figure 7:**
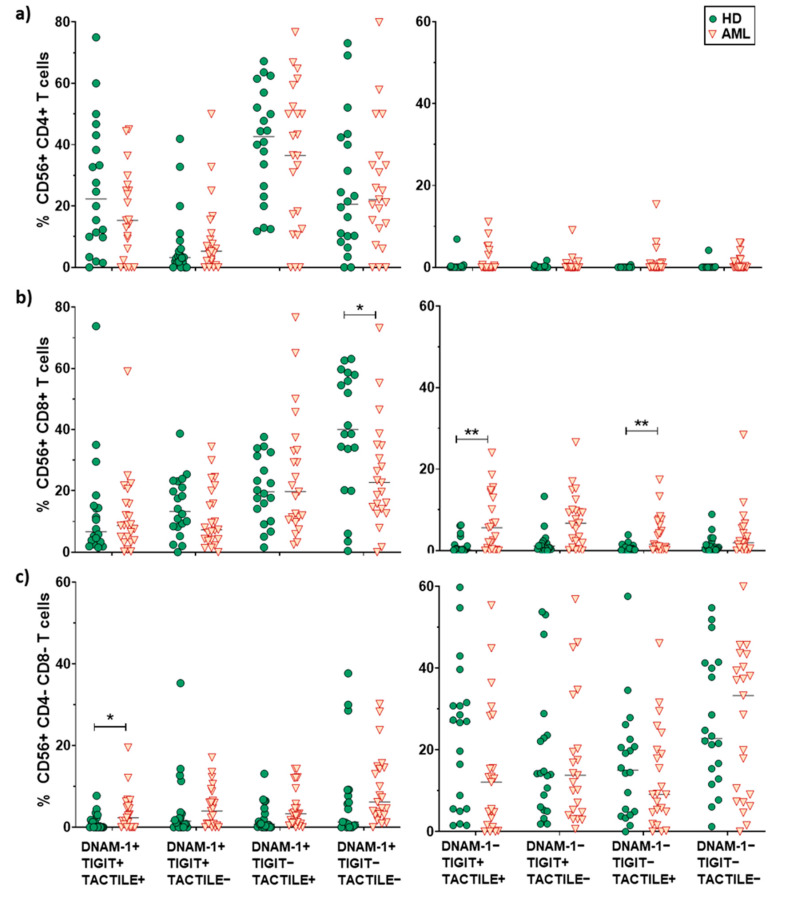
Analysis of DNAM-1, TIGIT and TACTILE co-expression in CD56+ T cell subsets. Eight different subpopulations can be observed according to the co-expression of DNAM-1, TIGIT and TACTILE. The distribution of these subsets in CD4+ T cells (**a**), CD8+ T cells (**b**) and CD4−CD8− T cells (**c**) in HD (*n* = 20) and AML patients (*n* = 23) is shown. The median values are indicated by a horizontal black line. Results were considered significant at * *p* < 0.05 and ** *p* < 0.01.

No differences in pie profiles were found between AML and healthy donors in any of the CD56+ T cells subsets (CD4+, CD8+ and DN) (Figure 5b and Figure 7). Nevertheless, in CD8+CD56+ T cells, DNAM-1+ TIGIT− TACTILE− cells were significantly reduced in AML patients compared with healthy donors (*p* = 0.047), whereas DNAM-1− TIGIT+ TACTILE+ cells (*p* = 0.01) and DNAM-1−TIGIT−TACTILE+ cells (*p* = 0.003) were increased (Figure 5b, middle panel, and Figure 7b). Finally, within the DN CD56+ T cell subset, we found an increase in the percentage of DNAM-1+TIGIT+TACTILE+ cells (*p* <0.05) in AML patients compared with healthy donors (Figure 5b, lower row, and Figure 7c). 

When comparing pie charts among subsets, significant differences were observed between CD4+ and CD8+ CD56− T cells both in healthy donors (*p* = 0.04) and in AML patients (*p* = 0.03). In CD56+ T cells, a different pie chart distribution between CD4+ and CD8+ T cells was observed in healthy donors (*p* = 0.004) but not in AML patients (*p* = 0.09). The distribution of the pie chart also differed between DN T cells and CD4+ or CD8+ T cells regardless of CD56 expression (*p* = 0.0001 in both healthy donors and AML patients) (Figure S2).

### 2.6. Survival Analysis

**Figure 8:**
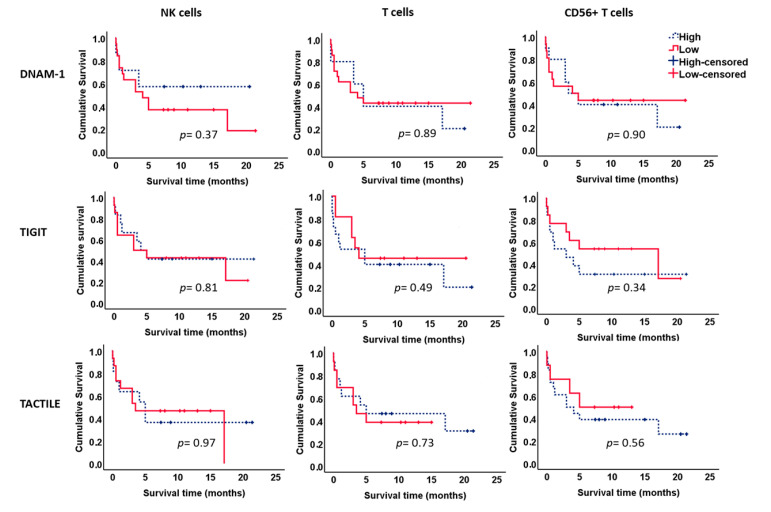
Kaplan–Meier survival analysis in AML patients. AML patients (*n* = 26) were stratified according to the expression of DNAM-1, TIGIT and TACTILE on NK cells, CD56− T cells and CD56+ T cells. ROC curves were used to calculate the optimal cut-off values. Blue dotted lines correspond to patients with high percentage of positive cells for the indicated receptor and red lines to patients with low percentage of positive cells. Censored patients are indicated as tick marks.

We further analyzed the effect of the expression of DNAM-1, TIGIT and TACTILE on the survival of AML patients (Figure 8). No significant differences were observed when each receptor was analyzed separately in NK cells or T cells.

**Figure 9:**
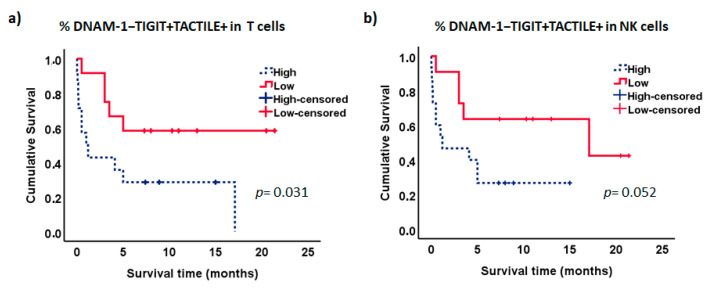
Kaplan–Meier survival analysis in AML patients. AML patients (*n* = 26) were stratified according to the co-expression of TIGIT and TACTILE on DNAM-1 negative CD56− T lymphocytes (**a**) and DNAM-1 negative NK cells (**b**). Blue dotted lines correspond to patients with high percentage of DNAM-1−TIGIT+TACTILE+ cells and red lines to patients with low percentage of DNAM-1−TIGIT+TACTILE+ cells. Censored patients are indicated as tick marks.

Interestingly, the analysis of the combined expression of these markers showed that patients with lower percentages of DNAM-1−TIGIT+TACTILE+ cells within the CD56− T cell subset had significantly longer survival than patients with higher percentages of T cells with this phenotype (*p* = 0.031, Figure 9a). Similarly, there was a trend for longer survival in those patients with lower percentages of NK cells co-expressing TIGIT and TACTILE in the absence of DNAM-1 (*p* = 0.052, Figure 9b). No significant differences were observed when the other receptor combinations were analyzed. No statistically significant relationships were found between the expression of the DNAM-1, TIGIT and TACTILE on NK or T cells, with other markers of poor prognosis such as the cytogenetic risk.

## 3. Discussion

The role in lymphocyte activation of pairwise receptors that share the same ligands has recently expanded due in part to the possibility to control inhibitory signaling by using blocking mAbs that regulate the immune response as it has been shown with the CD28/CTLA-4 axis in T cell activation. A novel candidate for checkpoint blockade is the inhibitory receptor TIGIT [21,23,24]. CD112 and CD155, members of the Nectin and Nectin-like families of molecules, respectively, are ligands for both inhibitory TIGIT and activating/costimulatory DNAM-1 receptors. Moreover, CD155 is also a ligand for the TACTILE receptor. Interestingly, CD112 and CD155 are frequently expressed in different types of cancer cells including myeloid leukemia blasts [9,21,23,24,25,26,27]. 

In this study, we firstly assessed the expression of DNAM-1, TIGIT and TACTILE in NK cells and T cells (CD4+, CD8+ and DN, stratified by the expression of CD56) in healthy donors and AML patients. In healthy individuals, our results show that DNAM-1 is expressed in most NK cells and CD4+ and CD8+ T cells (regardless of CD56 expression). We have also observed that TIGIT is present in a higher percentage of NK cells compared to T cells and that about half of NK and T cells express TACTILE. These results agree with previous data showing expression of these receptors in different lymphocytes subsets [13,28].

The role of DNAM-1 in recognition and lysis of AML leukemic blasts has been demonstrated in several models. Here, we have confirmed our previous results showing that NK cells from newly diagnosed AML patients have a reduced expression of DNAM-1 compared to healthy donors [5,6]. A decrease in the ratio DNAM-1+/DNAM-1− NK cells was also reported in patients diagnosed with Hodgkin lymphoma and diffuse large B-cell lymphoma, corresponding to low expression of DNAM-1 in NK cells with a terminally differentiated phenotype with reduced cytotoxic activity [29]. These results support that low expression of DNAM-1 on NK cells from AML patients contributes to the decreased NK cell cytotoxicity described in these patients. In the present study, we also show a downregulation of DNAM-1 expression on T cells in AML patients. The downregulation of DNAM-1 in AML patients is observed in both conventional CD56− T cells and CD56+ NKT-like cells. Our data on CD8+ T cells are consistent with previous findings showing a downregulation of DNAM-1 in CD8+ T cells in AML patients [15,16] as well as in metastatic melanoma [30]. Chronic exposure to DNAM-1 ligands has been proposed as a plausible mechanism involved in DNAM-1 downregulation [6,31]. Thus, the interaction of NK cells with AML blasts expressing DNAM-1 ligands, primarily CD112, results in the downregulation of DNAM-1 [5,6]. Similar results have been observed in CD8+ T cells from AML patients [15] and in NK cells after interaction with melanoma [32] or ovarian cell lines expressing CD155 [31]. On the contrary, the expression of TIGIT was not affected by coincubation of NK cells with target cells expressing CD112 or CD155 [33], whereas the expression of CD96 on the NK92 cell line was down-regulated CD96 after conjugation with the CD155-transfected Daudi cell line [34]. Besides, it has been shown that soluble human CD155, that binds with higher affinity to DNAM-1 than to TIGIT and CD96, affects DNAM-1-mediated NK cell degranulation in vitro and, in a mouse model, interferes with the DNAM-1-mediated cytotoxic activity of NK cells, and promotes murine melanoma progression [35]. Taken together, these results, from different experimental models, support that a decreased expression of DNAM-1 on NK cells is associated with a limited functional capacity and a defective cytotoxicity of target cell lines expressing CD112 or CD155.

Interestingly, we observed a higher percentage of conventional T cells expressing the inhibitory receptor TIGIT in AML patients compared with healthy donors, while no differences were found within NK or CD56+ T cells. TIGIT has been described as a marker of CD8+ T cell exhaustion in AML patients. TIGIT+ CD8+ T cells exhibit low production of cytokines that can be recovered by TIGIT knockdown, suggesting a role for TIGIT in the suppression of T cell anti-tumor responses [16]. The impact of TIGIT expression, analyzed by PCR, was studied in bone marrow samples after allo-stem cell transplantation in AML patients. High expression of TIGIT associated with poor overall and progression-free survival [36]. It has been shown, in experimental models, that TIGIT engagement with its ligands has an impact on both CD4+ and CD8+ T cell priming, suppressing effector function of chronically stimulated CD8+ T cells, and that the co-blockade of TIGIT and PD-1 synergistically enhances CD8+ T cell effector function [37]. In addition, TIGIT has higher affinity than DNAM-1 for CD155 binding, further contributing to NK and T cells dysfunction. Furthermore, TIGIT can also impair DNAM-1 function by disrupting DNAM-1 homodimerization [37]. Our results showing a significant downregulation of DNAM-1 and a higher expression of TIGIT in AML patients suggest that these alterations may be, at least in part, involved in the decreased effector functions of cytotoxic cells in these patients [38].

The role of TACTILE (CD96) as an immune checkpoint remains elusive with discrepant results in different experimental models. In our study, the individual analysis of this molecule showed no differences in AML patients compared to healthy individuals. An increased expression of TACTILE has been described in TILs in colorectal cancer in comparison with PBMCs [28]. Mittal et al. [39] have demonstrated that this receptor is expressed in mouse and human tumor-infiltrating CD8+ T cells and that blocking TACTILE/CD155 interaction alone or in combination with anti-PD-1 or anti-TIGIT enhances CD8+ T cell function. Thus, this receptor can act as an inhibitory receptor, supporting the interest for its clinical evaluation as a strategy for cancer immunotherapy. On the contrary, recent work by Chiang et al. [40] suggested that TACTILE can act as a costimulatory molecule for CD8+ T cell activation in vitro. Further analysis of antibodies used in the different settings may open light to clarify the role of TACTILE not only on NK cells but also on T cells. The analysis of patients with hepatocellular carcinoma show that intra-tumoral NK cells expressing TACTILE are functionally exhausted and TACTILE binding to CD155 on tumor cells reduces NK cell cytotoxicity and cytokine production. In addition, patients with higher numbers of TACTILE+ NK cells exhibit poorer clinical outcomes, suggesting an inhibitory function of TACTILE that contributes to immune escape of hepatocellular carcinoma [41].

In patients with gastric cancer, it has been shown that lower frequencies of tumor-infiltrating CD56+ NKT-like T cells are associated with poor survival. These tumor-infiltrating cells present lower effector function than those in non-tumor tissues and a reduced expression of CD69, DNAM-1 and NKG2D [42]. In this regard, our results show that co-expression of DNAM-1, TIGIT and TACTILE in CD56+ T cells is less affected by AML than their counterpart conventional CD56− T cells.

The double negative (CD4−CD8−) T cell subset represents a minor proportion of T cells, both in healthy donors and AML patients. Interestingly, the expression of DNAM-1 in DN T cells is significantly lower compared with the CD4+ and CD8+ T cell subsets both in healthy individuals and AML patients. Subsequently, all the DNAM-1+ subsets are diminished and DNAM-1− increased, irrespectively of the expression of TIGIT or TACTILE. Although it has been described that the DN T cell subset recognizes AML blasts preferentially in a TCR-independent manner that is, at least in part, mediated through NKG2D and DNAM-1 activating receptors [43], our results do not support a major role of DNAM-1 in the activation and recognition of target cells by DN T cells. The analysis of in vitro expanded DN T cells from healthy individuals has shown that these cells are cytotoxic against non-small-cell lung cancer cell lines, in vitro and in vivo in xenograft models, and express innate receptors such as NKG2D and DNAM-1, which can be up-regulated by IL-15, that are involved in the lysis of target cells [44]. A clinical trial (NCT03027102) is undergoing to analyze the effect of adoptive transfer of CD4−CD8− T cells in patients with high-risk AML. Our results on DN T cells in AML patients in comparison with healthy donors demonstrate differences in pie chart distribution with an increase in cells co-expressing DNAM-1, TIGIT and TACTILE. In addition, an increase in the percentage of cells expressing TIGIT and lacking DNAM-1 and TACTILE was observed in DN CD56− T cells in AML patients. The relevance of these changes in the functionality of these cells in AML blasts lysis requires further analysis.

TIGIT expression has been associated with poor clinical outcome in AML patients, and TIGIT+ CD8+ T cells represent exhausted cells with low production of cytokines that contribute to the functional T cell impairment observed in AML patients [16]. Wang et al. [15] also described the expansion in AML patients of a CD8+T cell subset expressing PD-1 and TIGIT and negative for DNAM-1 that was associated with poor prognosis such as failure to achieve remission after induction therapy or the presence of the FLT3-ITD mutation. Although we have not found statistically significant relationships between the expression of DNAM-1, TIGIT and TACTILE on NK or T cells with markers of poor prognosis such as the cytogenetic risk, the analysis of the NK and T cell subsets defined by the co-expression of DNAM-1, TIGIT and TACTILE shows that the expansion of the DNAM-1−TIGIT+TACTILE+ T cell subset is associated with lower survival in AML patients. In NK cells, high percentages of DNAM-1− TIGIT+ TACTILE+ cells also showed a tendency to be associated with reduced survival. Taken together, our results and those of others, support the hypothesis that the expansion of DNAM-1−TIGIT+ cells, in T and NK lymphocytes, contributes to the decreased recognition and lysis of AML blasts, previously observed in AML patients, that is also associated with poor patient survival. 

A limitation of this work is the heterogeneity of the AML patients and the difficulties to get enough numbers of patients in the different groups when stratifying them according to biomarkers of prognosis. Although our study shows that the percentage of DNAM-1−TIGIT+TACTILE+ T cells might be considered a prognostic factor for survival in AML patients, other factors such as age, cytogenetic profile or treatment procedures used will also affect patient survival. Thus, further studies in larger cohorts of patients are required to confirm the possible clinical relevance of this subset expansion as a biomarker of poor prognosis in AML patients. 

The analysis of DNAM-1, TIGIT and TACTILE demonstrating a different pattern of expression in NK and T cell subsets supports that the regulation of this axis of activating and inhibitory receptors that recognize proteins of the Nectin and Nectin-like families, frequently overexpressed in cancer, represents a new strategy to overcome tumor-induced suppression of both NK and T cells. The possibility to modulate this axis by increasing the expression of DNAM-1, e.g., using cytokines, or blocking the function of TIGIT represents novel therapeutic approaches. Checkpoint blockade using anti-TIGIT mAbs, alone or in combination with other therapies, may represent a good strategy for cancer therapy in AML. TIGIT mAbs are now included in clinical trials in cancer patients (for review see [21]). The effects of TIGIT blockade in non-small-cell lung cancer in combination with anti-PD-L1 mAbs (NCT04294810; NCT04256421) and in advanced metastatic solid tumors in combination with anti-PD-1 mAbs (NCT03119428; NCT04047862) are being analyzed. Furthermore, another inhibitory receptor, PVRIG (CD112R), that recognizes CD112 has been identified [45] and monoclonal antibodies to PVRIG are under study in clinical trials [21]. The pattern of DNAM-1, TIGIT and TACTILE co-expression in AML patients may constitute a biomarker for checkpoint blockade directed at inhibitory receptors that interact with CD112 and CD155 proteins. Our results support that the modulation of the DNAM-1/TIGIT/TACTILE axis can be a novel approach of immunotherapy to enhance both NK and T cell function against AML blasts.

## 4. Materials and Methods 

### 4.1. Patients and Samples

Thirty-six patients diagnosed with AML between 2017 and 2019 were included in the study and were followed up until the end of January 2020. The mean age of AML patients was 69.5 ± 16.5 (range 25–90) and 70% of AML patients were over 65 years old at the time of diagnosis. A total of 72.2% of AML patients were males and 27.8% were females (Table 1). Clinical characteristics of AML patients at the time of diagnosis are summarized in Table 2. Twenty healthy volunteers were also included in the analysis. The mean age of healthy volunteers was 59.8 ± 19 (range 18–91), 75% were males and 25% were females. 

The study was approved by the Institutional Ethical Committees of the University of Extremadura and Hospital San Pedro de Alcántara (Project SAF2017-87538-R; Cáceres, Spain) and Hospital Reina Sofía (Project PI16/01615 Córdoba, Spain) and the study was performed in accordance with the Declaration of Helsinki. Blood samples from healthy volunteers and adult patients with AML were obtained after informed consent.

Peripheral blood mononuclear cells (PBMCs) were obtained after density gradient centrifugation on Lymphoprep (STEMCELL Technologies, VAN, Canada). PBMCs were used for flow cytometry analysis and the rest of the cells were cryopreserved.

### 4.2. Antibodies

The following antibodies were used for flow cytometry analysis: anti-CD45 APC (Clone 2D1), anti-CD3-VioGreen (clone: REA613), anti-CD4-PE (Clone SK3BD) and anti-CD8-PerCP (Clone SK1BD) from BD Biosciences; anti-CD56-PE-Vio770, (clone: REA196), anti-CD4-VioBlue, (clone: VIT4), anti-DNAM-1 FITC (Clone DX11) and anti-CD96 (TACTILE)-APC (clone: REA195) from Miltenyi Biotec (Bergisch Gladbach, Germany) and anti-TIGIT-PE (MBSA43) from eBiosciences (San Diego, CA, USA).

### 4.3. Flow Cytometry

PBMCs were analyzed for the percentage of the different lymphocyte subsets by multiparametric flow cytometry. Samples were acquired in a MASCQuant (Miltenyi Biotec, (Bergisch Gladbach, Germany) flow cytometer and analyzed using FlowLogic (Inivai Technologies, Mentone, Victoria, Australia) and FlowJo (v10.0.7, TreeStar, Portland, OR, USA) software. Isotype-matched antibodies and fluorescence minus one (FMO) were used as controls. Lymphocytes were gated according to their expression of CD45, size and granularity (FSC vs. SSC). Within that gate, NK cells (CD3− CD56+), NKT-like cells (CD3+ CD56+) and conventional T cells (CD3+ CD56−) were gated by confronting CD56 vs. CD3. Individual gates (set based on fluorescence minus one and isotype-matched antibodies controls) for DNAM-1+, TIGIT+ and TACTILE+ cells were defined on each of these populations. FlowJo’s Boolean gating options were performed to analyze the co-expression of DNAM-1, TIGIT and TACTILE markers. The gating strategy is shown in Figure S3.

### 4.4. Statistical Analysis

For pie charts comparison we used SPICE permutation analysis [46]. SPICE stands for simplified presentation of incredibly complex evaluations and asks how often the difference observed between the samples represented in pie charts would happen simply by chance. 

The statistical analysis was performed using SPSS (version 22.0 statistical package (SPSS, Chicago, IL, USA). The GraphPad Prism program (version 7.0) was used for scatter graphs. Descriptive analysis of variables and normality tests were used. Mean differences were evaluated by Student’s *t* test and Mann–Whitney *U* test. Results were considered significant at *p* values < 0.05. The Kaplan–Meier estimator was used to perform the survival analysis. The differences were studied with the log-rank test.

## 5. Conclusions

This study confirms our previous results showing downregulation of DNAM-1 expression in NK cells from AML patients compared to healthy individuals. We also describe that this receptor is also decreased in conventional T cells and NKT-like CD56+ T cells. The reduced expression of DNAM-1 was associated with an increased percentage of DNAM-1− cells expressing TIGIT and/or TACTILE. The subpopulations of T lymphocytes that undergo most of the changes are CD56− T cells, predominantly CD8+ T cells. The subsets showing a reduction in DNAM-1 expression and an increase in TIGIT expressing cells are expanded in AML patients, supporting a shift in the balance to inhibition of lymphocyte function, limiting its ability to fight leukemia blasts. The role of TACTILE in NK and T cell activation remains elusive, but in the present study, TACTILE expression was not altered in NK cells and CD56+ T cells. A higher proportion of conventional CD56− T cells and NK cells lacking DNAM-1 and co-expressing TIGIT and TACTILE is associated with poor survival in AML patients. In conclusion, our results reveal a differential expression profile of DNAM-1, TIGIT and TACTILE in NK cells and T cell subsets in AML patients and highlight the importance of the combination of immunotherapeutic strategies aimed at improving lymphocyte activation by blockade of TIGIT checkpoint using anti-TIGIT mAbs in AML patients. The expansion of DNAM-1−TIGIT+TACTILE+/− cells in AML patients may constitute a biomarker of bad prognosis and the study of these subsets should be considered for checkpoint blockade-based therapy.

## Figures and Tables

**Table 1:** Percentage of NK cells and T cell subsets in healthy donors (HD) and acute myeloid leukemia (AML) patients.

Cell Subset	HD	AML	*p*
% NK cells (CD3− CD56+) *	26.3 ± 14.3	14.7 ± 13.9	0.003
% Conventional T cells (CD3+CD56−) *	44.3 ± 17.2	50.3 ± 17.2	0.53
CD4+ T cells **	53.4 ± 16.9	54.1 ± 15.4	0.89
CD8+ T cells **	35.9 ± 13.5	35.4 ± 13.3	0.90
DN T cells **	7.1 ± 4.4	7.7 ± 4.4	0.63
DP T cells **	0.6 ± 0.2	0.6 ± 0.4	0.62
% NKT-like cells (CD3+CD56+) *	2.7 ± 3.1	4.7 ± 5.1	0.13
CD4+ T cells **	15.6 ± 14.5	8.3 ± 12.4	0.08
CD8+ T cells **	59.9 ± 17.8	62.1 ± 20.6	0.71
DN T cells **	17.0 ± 11.0	22.3 ± 15.6	0.21
DP T cells **	1.0 ± 1.1	0.8 ± 1.3	0.58

* Referred to lymphocyte gate. ** Referred to the corresponding subset. HD: *n* = 20. AML: *n* = 36; analysis of CD4, CD8 on AML *n* = 23. DP: CD4+CD8+ double positive; DN: CD4−CD8− double negative.

**Table 2:** Clinical characteristics of patients with AML.

Characteristic	Value
No. Patients	36
Age	
Median (Range)	74.5 (25–90)
Mean (SD)	69.5 ± 16.5
Sex no. (%)	
Male	26 (72.2%)
Female	10 (27.8%)
FAB classification no. (%)	
M0	0%
M1	6 (16.7%)
M2	3 (8.3%)
M3	1 (2.8%)
M4	5 (13.9%)
M5	9 (25%)
M6	1 (2.8%)
M7	1 (2.8%)
Secondary AML no. (%)	6 (16.7%)
AML classification not available	4 (11.1%)
Cytogenetic risk no. (%)	
Favorable	4
Intermediate	17
Adverse	8
Not available	7

## Supplementary Materials

The following are available online at https://www.mdpi.com/2072-6694/12/8/2171/s1, Figure S1: Comparison of co-expression patterns of DNAM-1, TIGIT and TACTILE among NK cells, CD56− and CD56+ T cell subsets, Figure S2: Comparison of co-expression patterns of DNAM-1, TIGIT and TACTILE in T cell subsets, Figure S3: Gating strategy used for the analysis of DNAM-1, TIGIT and TACTILE expression on NK cells, CD56− T cells and CD56+ T cells.
