# Supplementary material for: Characterization of the DNAM-1, TIGIT and TACTILE Axis on Circulating NK, NKT-Like and T Cell Subsets in Patients with Acute Myeloid Leukemia
Source: Cancers (Basel). 2020 Aug 5;12(8):2171. doi: 10.3390/cancers12082171 (PMC7464787; doi:10.3390/cancers12082171)
Supplement: Supplementary file 1 [file cancers-12-02171-s001.pdf]

Supplementary Materials: Characterization of the DNAM-1, TIGIT and TACTILE Axis on Circulating NK, NKT-Like and T Cell Subsets in Patients with Acute Myeloid Leukemia

Isabel Valhondo, Fakhri Hassouneh, Nelson Lopez-Sejas, Alejandra Pera, Beatriz Sanchez-Correa, Beatriz Guerrero, Juan M. Bergua, Maria Jose Arcos, Helena Bañas, Ignacio Casas-Avilés, Joaquin Sanchez-Garcia, Josefina Serrano, Carmen Martin, Esther Duran, Corona Alonso, Rafael Solana and Raquel Tarazona

a) Healthy donors

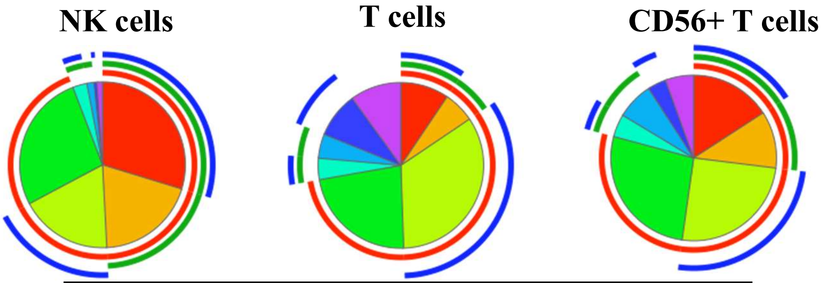

Pie Category Test Results			
**Pie #**	**NK Cell**	**T Cells**	**CD56+ T Cells**
NK cell		<0.0001	0.0262
T cells	<0.0001		0.0113
CD56+ T cells	0.0262	0.0113	

b) AML patients

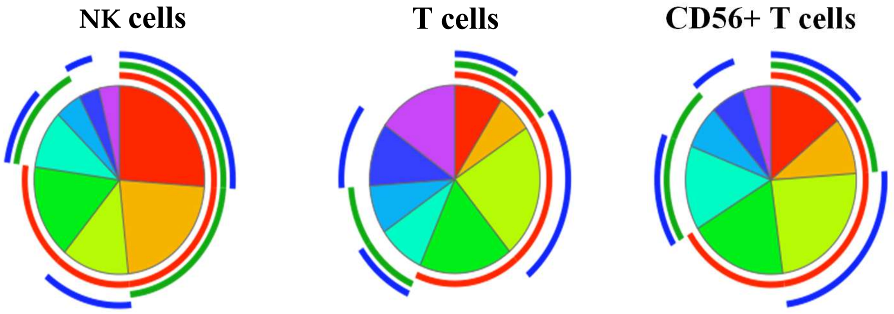

Pie Category Test Results			
**Pie #**	**NK Cell**	**T Cells**	**CD56+ T Cells**
NK cell		<0.0001	<0.0001
T cells	<0.0001		0.0170
CD56+ T cells	<0.0001	0.0170	

Figure S1. Comparison of co-expression patterns of DNAM-1, TIGIT and TACTILE among NK cells, CD56− and CD56+ T cell subsets. Co-expression patterns (pie charts) were analyzed in NK cells, conventional CD56− T cells and CD56+ NKT-like cells in (**a**) healthy donors ($n = 20$) and (**b**) AML patients ($n = 30$). The tables below the pie charts correspond to the results of the statistical analysis.

a) Healthy donors

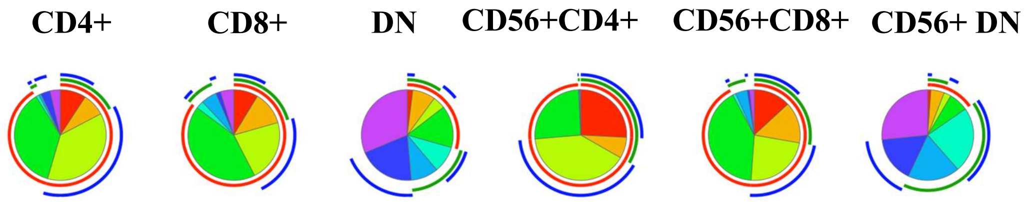

Pie Category Test Results						
**Pie #**	**CD4+**	**CD8+**	**DN**	**CD56+CD4+**	**CD56+CD8+**	**CD56+ DN**
CD4+		0.0365	<0.0001	0.0198	0.0629	<0.0001
CD8+	0.0365		<0.0001	0.0003	0.7279	<0.0001
DN	<0.0001	<0.0001		<0.0001	<0.0001	0.0128
CD56+CD4+	0.0198	0.0003	<0.0001		0.0035	<0.0001
CD56+CD8+	0.0629	0.7279	<0.0001	0.0035		<0.0001
CD56+ DN	<0.0001	<0.0001	0.0128	<0.0001	<0.0001	

b) AML patients

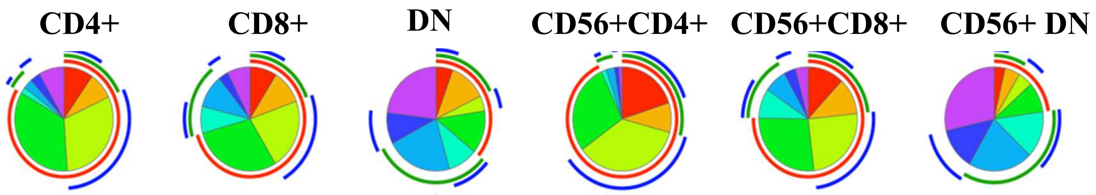

Pie Category Test Results						
**Pie #**	**CD4+**	**CD8+**	**DN**	**CD56+CD4+**	**CD56+CD8+**	**CD56+ DN**
CD4+		0.0310	<0.0001	0.0855	0.0664	<0.0001
CD8+	0.0310		<0.0001	0.0121	0.7840	<0.0001
DN	<0.0001	<0.0001		<0.0001	<0.0001	0.2585
CD56+CD4+	0.0855	0.0121	<0.0001			<0.0001
CD56+CD8+	0.0664	0.7840	<0.0001	0.0959		<0.0001
CD56+ DN	<0.0001	<0.0001	0.2585	<0.0001	<0.0001	

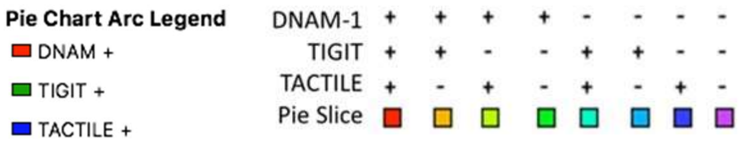

Figure S2. Comparison of co-expression patterns of DNAM-1, TIGIT and TACTILE in T cell subsets. Co-expression patterns (pie charts) were analyzed in CD4+, CD8+, and DN (CD4- CD8-, double negative) T cell subsets distributed according to CD56 expression in (**a**) healthy donors ($n = 20$) and (**b**) AML patients ($n = 23$). The tables below the pie charts correspond to the results of the statistical analysis.

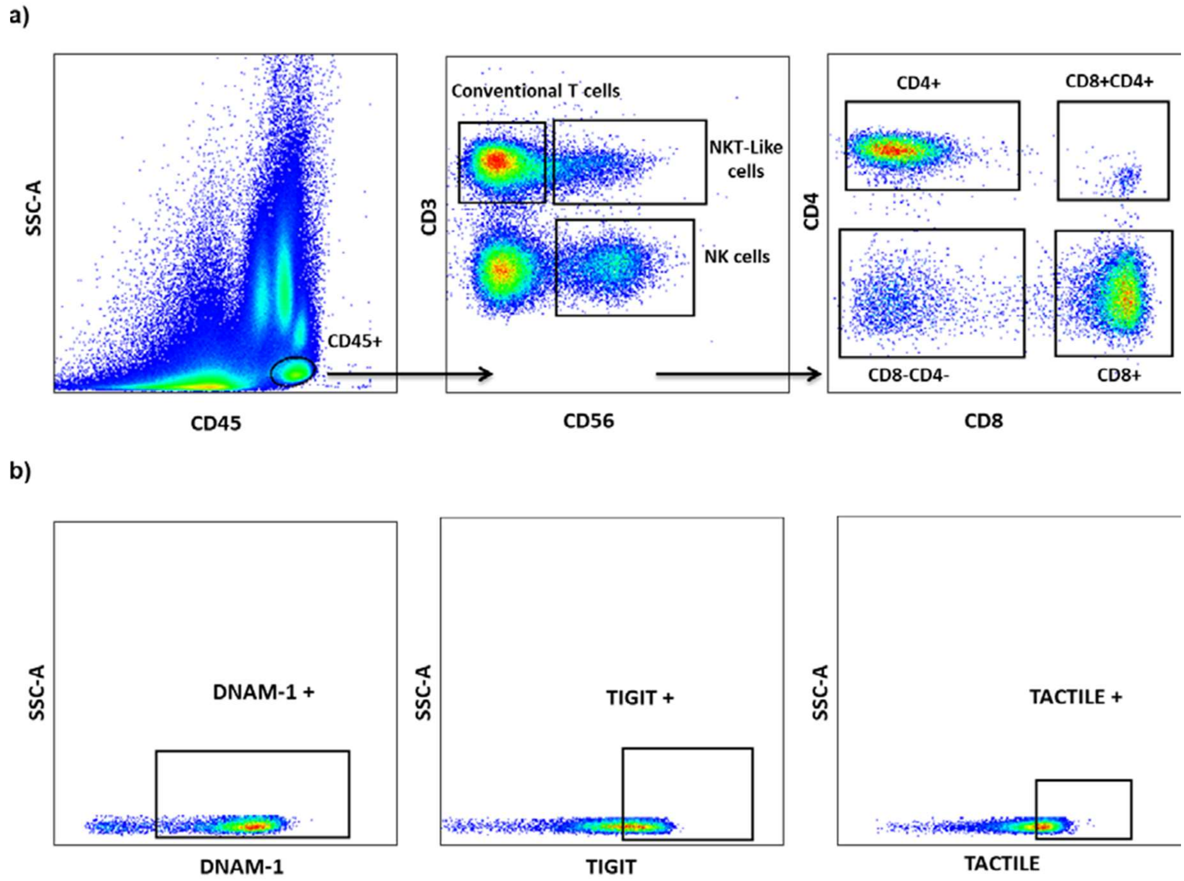

Figure S3. Gating strategy used for the analysis of DNAM-1, TIGIT and TACTILE expression on NK cells, CD56− T cells and CD56+ T cells. (**a**) Lymphocytes were selected according to their expression of CD45, size and granularity (FSC vs. SSC), subsequently NK cells (CD3− CD56+), NKT-like cells (CD3+ CD56+) and conventional T cells (CD3+ CD56−) were gated by confronting CD56 vs CD3, and followed by the identification of CD4+, CD8+, CD4-CD8- cells (DN: Double negative), CD4+CD8+ cells (DP: Double positive). (**b**) Subsequently, the expression DNAM-1, TIGIT and TACTILE was determined within NK cells (CD3− CD56+), NKT-like cells (CD3+ CD56+) and conventional T cells (CD3+ CD56−) using Fluorescence minus one (FMO) and isotype controls. Figure shows an example for NK cell.

© 2020 by the authors. Licensee MDPI, Basel, Switzerland. This article is an open access article distributed under the terms and conditions of the Creative Commons Attribution (CC BY) license (http://creativecommons.org/licenses/by/4.0/).
